# Supplementary material for: ACIDES: on-line monitoring of forward genetic screens for protein engineering
Source: Nat Commun. 2023 Dec 26;14:8504. doi: 10.1038/s41467-023-43967-9 (PMC10751290; doi:10.1038/s41467-023-43967-9)
Supplement: Supplementary file 1 — Supplementary Information [file 41467_2023_43967_MOESM1_ESM.pdf]

Supplementary information for ACIDES: on-line monitoring of forward genetic screens for protein engineering

Takahiro Nemoto,[1, 2, 3, *] Tommaso Ocari,[1] Arthur Planul,[1] Muge Tekinsoy,[1] Emilia A. Zin,[1] Deniz Dalkara,[1, †] and Ulisse Ferrari[1, ‡]

[1] *Institut de la Vision, Sorbonne Université, INSERM, CNRS, 17 rue Moreau, 75012, Paris, France*
[2] *Graduate School of Informatics, Kyoto University, Yoshida Hon-machi, Sakyo-ku, Kyoto, 606-8501, Japan*
[3] *Premium Research Institute for Human Metaverse Medicine (WPI-PRIMe), Osaka University, Suita, Osaka 565-0871, Japan*

* nemoto.takahiro.prime@osaka-u.ac.jp
† deniz.dalkara@inserm.fr
‡ ulisse.ferrari@inserm.fr

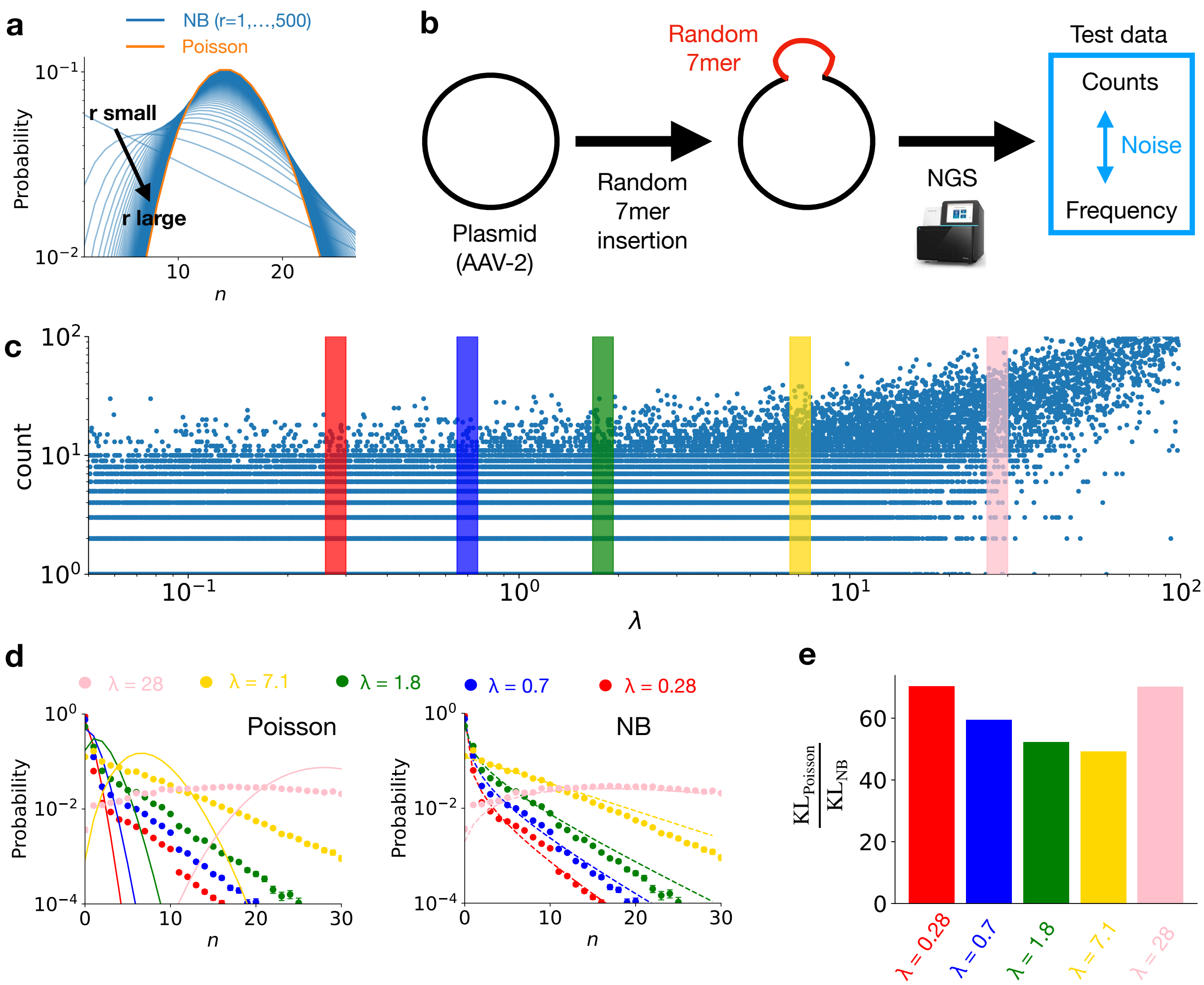

FIG. S1. **Negative binomial model accounts for NGS count noise better than Poisson model.** **(a)** The poisson distribution (orange) and the negative binomial distribution (tableau blue) with the expected value $\lambda = 15$. The dispersion parameter r for the negative binomial distribution is set to $1, 2, ..., 500$. The negative binomial distribution generalizes the Poisson distribution, allowing for large variances by decreasing r. It converges to the Poisson distribution in the large r limit. **(b)** In order to test the predictive ability of the negative binomial distribution, we performed the following experiment. Using a random peptide (of size 21 corresponding to a 7mer) as a barcode, we first barcoded a plasmid extracted from adeno-associated virus 2 (AAV2) wild type. The obtained 7-mer inserted library was then sent to NGS facility and the corresponding barcoded region was sequenced. Since these 21 nucleotides of barcode are randomly and independently generated, we can use a position weight matrix model to predict the frequency of each variant in the sample. Comparing the predicted frequency with the actual NGS reads, we investigate the noise distribution of NGS counts. **(c)** The graph showing the obtained counts (n) against the predicted frequencies multiplied by the total NGS reads (λ), where each point corresponds to a variant. We observe that the counts are largely dispersed. **(d)** The probability distribution of counts for a fixed value of λ together with the model predictions by Poisson distribution (left) and by the negative binomial distribution (right). The probability distribution is estimated in the following way: (i) picking up all the variants within 5 different colored rectangles in the panel (c). (ii) Using the variants corresponding to each rectangle, we then make a histogram of counts, which is plotted in the panel (d) as dots. For the Poisson prediction, we simply use the Poisson distribution with the mean $\lambda = 0.28, 0.7, 1.8, 7.1, 28$. For the negative binomial prediction, for each value of λ, we infer the dispersion parameter r via a maximum likelihood inference and fitted a power-law function $r = \beta\lambda^{\alpha}$ to the obtained estimations. The result is $r = 0.21\lambda^{0.744}$. Using this relation, the negative binomial distribution is then plotted for each $\lambda = 0.28, 0.7, 1.8, 7.1, 28$. **(e)** Comparison of predictive abilities between the poisson model and the negative binomial model. To quantify the predictive ability of each model, we use Kullback-Leibler divergence (KL) defined as $\mathrm{KL} = \sum_n P_{\mathrm{data}}(n) \log(P_{\mathrm{data}}(n)/P_{\mathrm{model}}(n))$. The ratio between KL for the poisson model and KL for the negative binomial model is plotted for each value of λ. $\mathrm{KL}_{\mathrm{Poisson}}$ itself is 0.37, 0.59, 0.87, 1.59, 3.29 for $\lambda = 0.28, 0.7, 1.8, 7.1, 28$, while $\mathrm{KL}_{\mathrm{NB}}$ is 0.0053, 0.0099, 0.017, 0.032, 0.047. Source data are provided as a Source Data file.

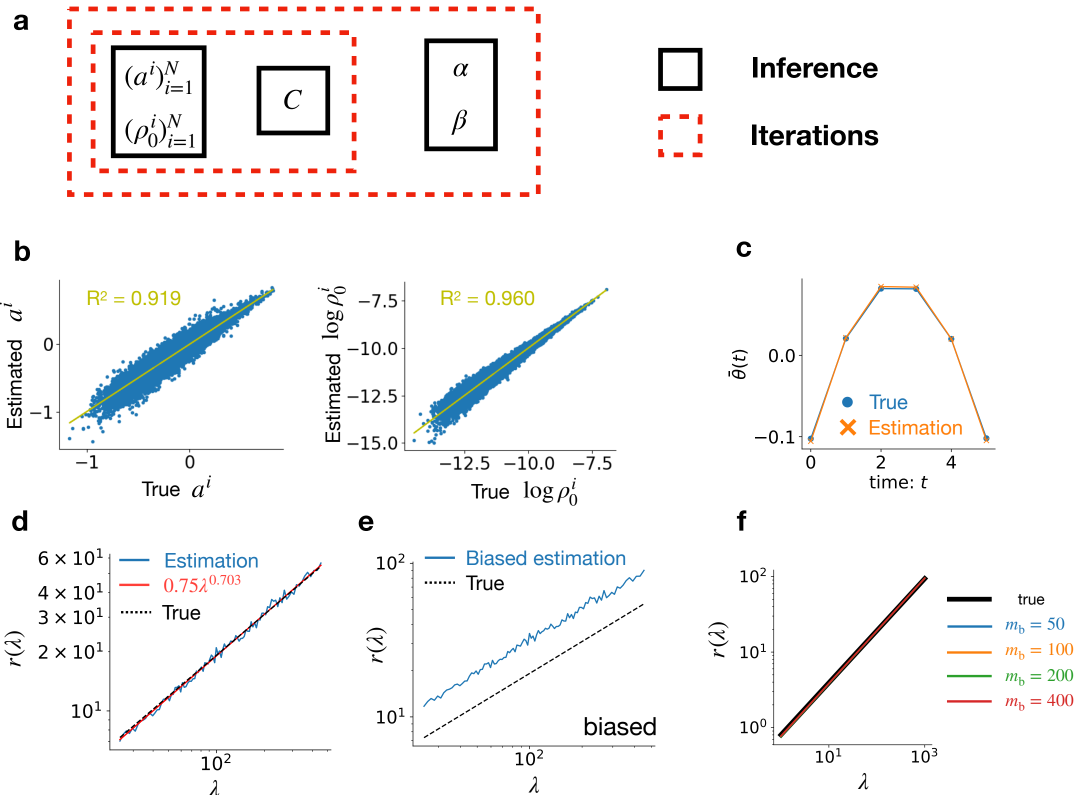

FIG. S2. **Inference algorithm and synthetic teacher-student examples.** **(a)** graphical illustration of the inference algorithm with its double loop structure. The internal loop accounts for the parameters of the exponential model (see Materials and Methods) and iterates between the inference of $(a^i, \rho_0^i)_{i=1}^M$ and C, while the external loop iterates between this internal loop and the inference of the negative binomial parameters (α, β). For the internal loop, we first infer $(a^i, \rho_0^i)_{i=1}^M$ for a fixed C, α, β by maximizing the likelihood function (2). C^* is then calculated from the obtained $(a^i, \rho_0^i)_{i=1}^M$ via $C^* = 1/\sum_i \rho_0^i \exp(a^i t)$. The linearly increasing part of $\log C^*$ is next subtracted as $\log C^* - (x^* t + y^*)$, where $x^*, y^* = \mathrm{argmax}_{x,y} \sum_t [\log C - xt - y]^2$ (fixing a gauge). The obtained quantity is $\log C$ for the next iteration. For the external loop, to obtain (α, β), we first infer the dispersion parameter r for a list of different values of λ. To do so, for a fixed value of λ, we select a set of index i and the time t by the condition $\lambda < \rho_t^i N_t < \lambda + \epsilon$ (with a small parameter ϵ). Only using these i and t, we then maximize the likelihood function (2) and determine the value of $r(\lambda)$. After obtaining the function $r(\lambda)$ for several values of λ, we fit a linear function $r(\lambda) = \alpha\lambda^\beta$ to it and determine α and β. **(b,c,d)** Ground truth comparisons for the synthetic data-rich dataset (Fig. 1e) after 30th iterations demonstrates that the algorithm can recover the generating parameters. In the panel (b), we plot the estimated parameters $(a^i)_{i=1}^M$ (left) and $(\log \rho_0^i)_{i=1}^M$ (right) against their ground truths. The coefficient of determination R^2 is also shown. In the panel (c), the normalization coefficient $(\bar{\theta}(t) \equiv \log C)$ is plotted together with its ground truth. In the panel (d), the estimated $r(\lambda)$ with a fitted line $\beta\lambda^\alpha$ and its ground truth are shown. **(e)** While estimating $r(\lambda)$ for a fixed λ, maximizing the likelihood function (2) results in a biased estimation as shown in the panel (e). For fixing this, we generate a synthetic data probe using the current estimation of α_0 and β_0 with $(\rho_t^i)_{i,t}$ and use it to unbias the r estimation. More precisely, denoting the biased estimation by $r_{\mathrm{bias}}(\lambda)$ (panel (e)) and the estimation of the r in the probe by $r_1(\lambda)$, the unbiased estimator plotted in the panel (d) is obtained as $r(\lambda) = r_{\mathrm{bias}}(\lambda)\beta_0\lambda^{\alpha_0}/r_1(\lambda)$. **(f)** To determine α and β, we use only representative variants, as described in Materials and Methods. For the representative variants, we select the variants whose total counts are larger than a threshold value m_{b}. In (F), by using the synthetic data, we show that the inference results (α and β) are robust against the change of this parameter m_{b}. Source data are provided as a Source Data file.

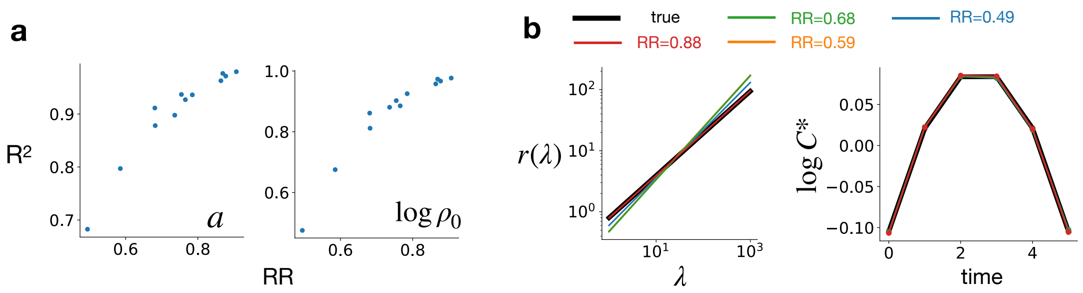

FIG. S3. **RR offers a proxy for the accuracy of the estimated scores.** Here we use synthetic datasets, ranging from data-poor to data-rich regimes, to show that the empirical quantity RR correlates with our ability to recover the true values of scores a^i and initial frequencies ρ_0^i. For this, we generate synthetic datasets with different values of total NGS reads N and number of variants M: $(N, M) = (10^7, 5\times10^4)$; $(8\times10^6, 5\times10^4)$; $(6\times10^6, 5\times10^4)$; $(4\times10^6, 5\times10^4)$; $(2\times10^6, 5\times10^4)$; $(10^6, 5\times 10^4)$; $(10^7, 10^6)$; $(8\times10^6, 10^6)$; $(6\times10^6, 10^6)$; $(4\times10^6, 10^6)$; $(2\times10^6, 10^6)$; $(10^6, 10^6)$. In these datasets, the parameters (α, β) are the same as those used in Fig.1e, f. **(a)** Coefficients of determination R^2 between inferred and true values for a^i (left) and ρ_0^i (right) are plotted against RR. This demonstrates the correlation between R^2 and RR. **(b)** The inference of $r(\lambda)$ and C^* is robust across all synthetic datasets. Source data are provided as a Source Data file.

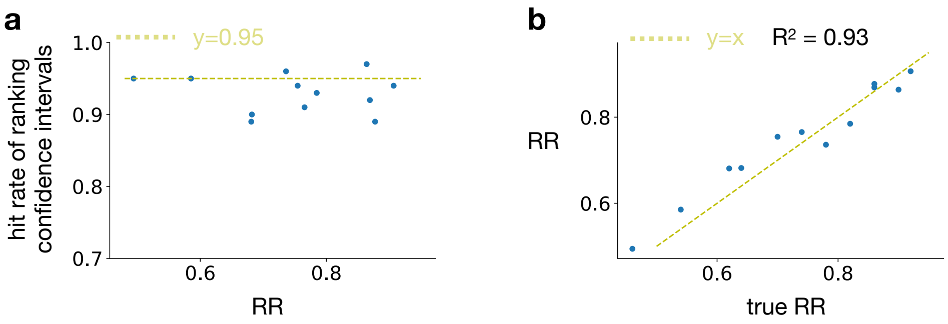

FIG. S4. **Ranking CI and rank robustness are accurately estimated also in the data-poor regime. (a)** For all the synthetic datasets introduced in Fig. S3. hit rates of the confidence interval of ranking graphs are plotted against their RR, where the hit rate is defined as the number of true ranking (red crosses in Fig.1e, f for example) that are within the 95%-confidence intervals (green lines in Fig.1e, f), divided by 50. The hit rates fluctuate around 0.95 as expected, demonstrating the quality of our estimation of ranking CI. **(b)** Rank robustness (RR) estimated from inferred parameters is plotted against the true value (ground truth) for the synthetic datasets. The obtained high coefficient of determination shows that ACIDES can estimate RR also in the data-poor regime. Source data are provided as a Source Data file.

TABLE S1. Additional information on experiments with multiple time-points

Label	A	B	C	D	E	F	G
Replicates	2 x 3	2 x 3	1 x 1	2 x 1	1 x 1	2 x 1	1 x 1
α	0.59	0.87	0.15	0.01	0.15	0.77	0.03
β	0.97	0.24	5.10	23.12	0.19	0.28	0.16

Additional information on the experimental datasets of table I. In *Replicates*, $x \times y$ means that there are x replicates that do not share the same initial library, each of which has y technical replicates (that shares the same initial library). α and β are the inferred model parameters.

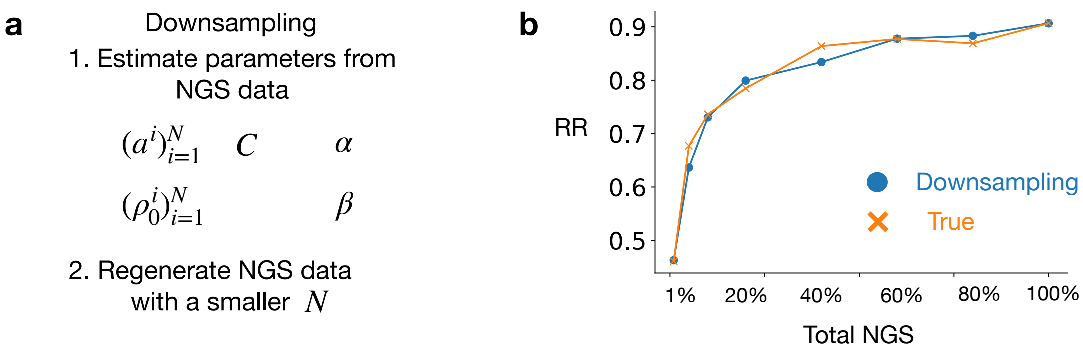

FIG. S5. **Downsampling NGS counts.** **(a)** For a given dataset of a DE experiment, we downsample the data, *i.e.*, create a synthetic dataset that corresponds to the dataset of the same DE experiments but with smaller values of the total NGS reads. For this, we first estimate the parameters of ACIDES from the dataset. We then generate synthetic NGS data using the likelihood function (2) with smaller numbers of the total NGS reads. For example, if we downsample the data to 40%, we set N_t to be $0.4N_t$. We then estimate RR using ACIDES for this downsampled dataset without reinferring α, β. **(b)** We show the validity of this down sampling method on the synthetic data. The parameters for the synthetic data are $(N_t, M) = (10^7, 5\times10^5)$, $(\alpha, \beta) = (0.69, 0.8)$ and $(a^i, \log\rho_0^i)_{i=1}^M$ generated from the normal distribution with the expected values (-1,1) and the standard deviation (0.25, 1). We plot RRs obtained from this downsampling method (blue circles) and from a standard sampling method (orange crosses) as a function of the total number of NGS (where 100% means the original data). Here the standard sampling method means using ACIDES directly on the dataset with the total number of NGS $0.01xN_t$, where x is the percentage of the total NGS reads (x-axis in the panel (b)). We observe that our downsampling method estimates well the RR. Source data are provided as a Source Data file.

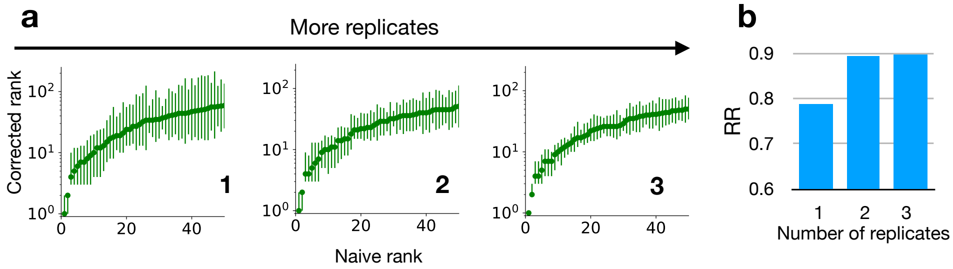

FIG. S6. **Multiple replicates can be combined to increase RR.** We use the first 4 rounds of Data-A (so that RR is relatively small for a single replicate), and perform ACIDES for each replicate. **(a)** Ranking graphs for one (left), two (middle), three (right) replicates. The corrected ranks are shown with 95%-CI, estimated using 3000 bootstrap samples (Methods). To combine variant scores of two replicates (denoted by a_1, a_2 with standard deviation δa_1, δa_2), we use $(a_1\delta a_2^2+a_2\delta a_1^2)/(\delta a_1^2+\delta a_2^2)$ for the combined score and $\sqrt{\delta a_1^2\delta a_2^2}/(\delta a_1^2+\delta a_2^2)$ for the combined standard deviation. **(b)** RR for the three cases. Source data are provided as a Source Data file.

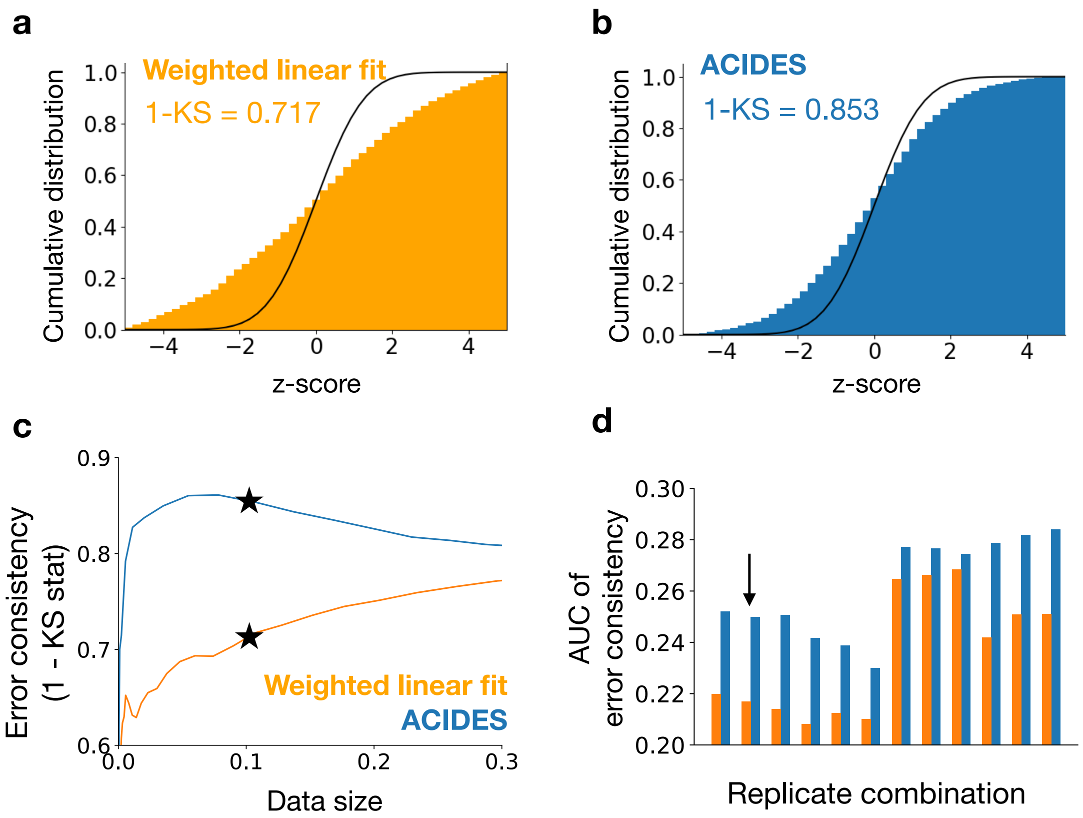

FIG. S7. **ACIDES outperforms previous methods in the estimation of score's statistical errors.** Using replicates in Data-A and Data-B (Table I), we study the consistency of error bars in ACIDES and in Enrich2. Denoting by a_1, a_2 the scores of a variant in replicate 1 and 2 (similarly by δa_1, δa_2 the standard deviations of the scores), we study the following quantity $z = (a_1 - a_2)/\sqrt{\delta a_1^2 + \delta a_2^2}$ and compute the histogram of this quantity over different variants. Under the assumption that both scores are distributed following the normal distribution, this obtained histogram is approximated by the standard normal distribution. **(a,b)** Cumulative distributions of the histograms for Enrich2 (a) and ACIDES (b) together with the cumulative standard normal distribution. We use $1-$ Kolmogorov–Smirnov (KS) statistics (the maximum distance between two distributions measured in the y-direction) to quantify the distance between the histogram and the normal distribution. **(c)** To study $1 - \text{KS}$ more systematically, we introduce a threshold for the score statistical errors (Materials and Methods) by which we reduce the amount of data. For each fraction of the data, we estimate $1 - \text{KS}$ and plot them in the panel (c). The stars in the panel correspond to panels (a) and (b). **(d)** Finally, using all possible combinations of technical replicates in Data-A and Data-B, we compare ACIDES and Enrich2. We compute the area under curve (AUC) of $1 - \text{KS}$ (the panel (c)) for all combinations. ACIDES always shows better performance than Enrich2. The arrow in the panel (d) indicates the replicate combination used in the panels (a-c). Source data are provided as a Source Data file.

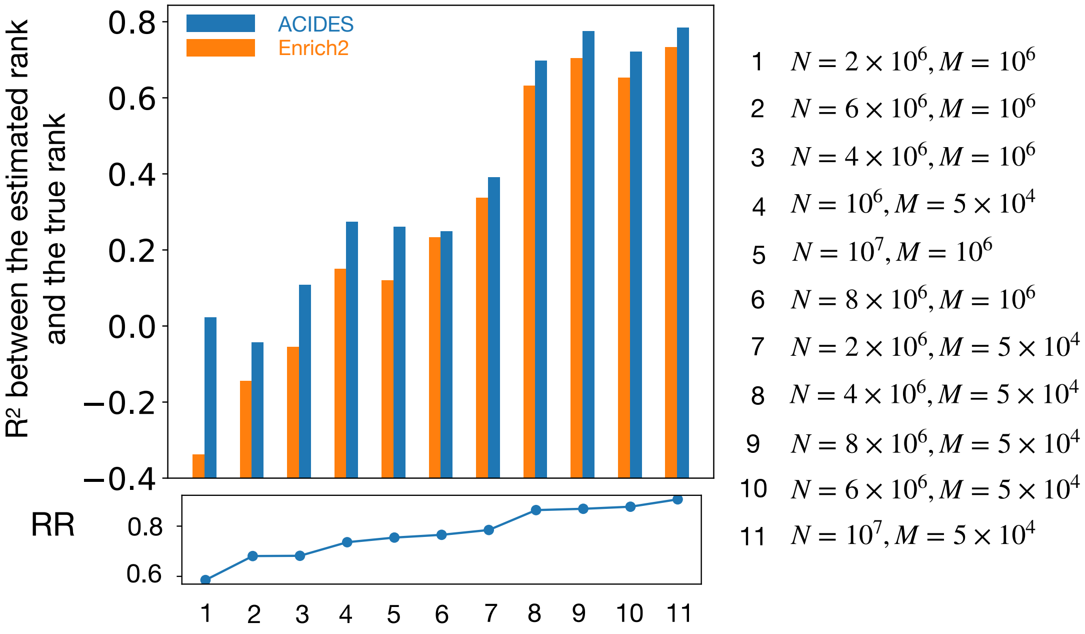

FIG. S8. **Comparison between ACIDES and Enrich2 using synthetic dataset.** Using synthetic dataset with different values of total NGS reads N and number of variants M, we estimate the R^2 values between the ground truth and either ACIDES corrected rank (green) or the rank estimated from the application of Enrich2 (red). The better performance of ACIDES becomes more pronounced as RR decreases. Source data are provided as a Source Data file.
